# Multi-omics-based subtyping of melanoma suggests distinct immune and targeted therapy strategies

**DOI:** 10.3389/fimmu.2025.1601243

**Published:** 2025-06-12

**Authors:** Changchang Li, Xiaoqiong Lin, Jinhui Wang, Qiaochu Zhou, Fangfang Feng, Jie Xu

**Affiliations:** Wenzhou Hospital of Integrated Traditional Chinese and Western Medicine, Wenzhou, Zhejiang, China

**Keywords:** melanoma, multi-omics integration, molecular subtypes, immune checkpoint therapy, in-silico drug screening

## Abstract

**Background:**

Melanoma is a highly heterogeneous malignancy with diverse molecular and clinical behaviors. A precise molecular classification is critical for improving prognostic assessment and guiding personalized therapy.

**Methods:**

We performed an integrative multi-omics analysis of skin cutaneous melanoma using data from The Cancer Genome Atlas (TCGA) and validated our findings in independent cohorts. Multi-layered data, including transcriptomic, genomic, epigenetic, and immune landscape profiles, were analyzed using unsupervised clustering and machine learning approaches to define molecular subtypes. Functional assays and in silico drug screening were employed to explore subtype-specific vulnerabilities.

**Results:**

Three robust molecular subtypes (CS1, CS2, CS3) were identified, each with distinct genomic alterations, tumor microenvironment characteristics, and clinical outcomes. The CS2 subtype was immunologically “hot,” characterized by high tumor mutational burden (TMB), elevated neoantigen load, strong immune infiltration, and activated IFN-γ signaling. CS2 tumors showed significant enrichment of immune checkpoint gene expression and were associated with favorable response to anti-PD-1 therapy in external validation cohorts. In contrast, CS1 and CS3 were immunologically “cold” with immune exclusion, high chromosomal instability, and activation of oncogenic pathways linked to immune evasion. Transcriptomic drug sensitivity modeling suggested that CS1 and CS3 may benefit from HSP90 or MEK inhibitors. Moreover, COL11A2 was identified as a subtype-enriched oncogenic driver predominantly expressed in CS1/CS3, and its silencing impaired tumor cell proliferation, invasion, and epithelial–mesenchymal transition (EMT) features.

**Conclusions:**

This study presents a refined multi-omics classification of melanoma that reveals biologically and clinically distinct subtypes with divergent immune and therapeutic profiles. It offers a framework for subtype-specific treatment strategies, and identifies COL11A2 as a potential target in immune-cold melanomas.

## 1 Introduction

Melanoma is a highly aggressive and deadly skin cancer, causing around 57,000 deaths globally each year (1). Despite advancements in treatments such as immune checkpoint inhibitors targeting PD-1 or CTLA-4, and therapies for BRAF/MEK mutations, the prognosis for metastatic melanoma remains poor, with a five-year survival rate of just 32% (2, 3). A major obstacle to effective treatment is the pronounced intra-tumoral heterogeneity of melanoma, which drives therapeutic resistance and promotes aggressive disease progression (4).

Emerging research has unveiled the extraordinary adaptability and phenotypic diversity of melanoma cells. Single-cell RNA sequencing studies have identified four predominant transcriptional subtypes (5, 6): (1) an invasive, poorly differentiated population with suppressed MITF and elevated AXL levels; (2) a stem-like subset resembling neural crest precursors, distinguished by upregulated developmental markers; (3) a differentiated melanocytic lineage expressing pigment synthesis and melanocyte-specific genes; and (4) an intermediate phenotype displaying mixed features. These subpopulations dynamically interact within tumors and undergo selective pressure during treatment, frequently enriching for therapy-resistant, metastatic variants (7). Nevertheless, the precise molecular controls orchestrating these transitions and their distinct roles in disease aggressiveness remain elusive.

Beyond transcriptional plasticity, melanoma exhibits extensive genetic and phenotypic heterogeneity (8). For instance, tumor subpopulations can be stratified based on the expression of microphthalmia-associated transcription factor (MITF), with MITF-high and MITF-low states contributing to both intra- and intertumoral heterogeneity (9). Additionally, genetic diversity—such as spatial variations in BRAF mutations—can influence responses to targeted therapies (10), underscoring the need for comprehensive molecular profiling to guide treatment decisions. Critically, this heterogeneity leads to divergent therapeutic responses, as resistant subclones evade treatment and drive disease relapse.

To address these challenges, we performed a comprehensive multi-omics integration of cutaneous melanoma to delineate robust molecular subtypes and elucidate the biological drivers of tumor heterogeneity. Using a Bayesian integrative framework, we identified novel subtypes with distinct molecular, clinical, and immunological features. Our findings provide a refined molecular taxonomy of melanoma with clear prognostic and therapeutic implications, offering a foundation for improved patient stratification and personalized immunotherapy strategies.

## 2 Materials and methods

### 2.1 Multi-omics discovery cohort from TCGA-SKCM

We developed a multi-omics discovery cohort for cutaneous melanoma using data from The Cancer Genome Atlas (TCGA-SKCM) (11). This included 421 primary or metastatic tumor samples with matched transcriptomic, DNA methylation, somatic mutation, copy number alteration (CNA), clinical, and survival data. Gene expression profiles (n = 453) were obtained from UCSC Xena (http://xena.ucsc.edu/) and initially expressed as fragments per kilobase million (FPKM). These were then converted to transcripts per kilobase million (TPM) to enable cross-sample comparison. Both mRNAs and long non-coding RNAs (lncRNAs) were analyzed, with lncRNA annotations derived from the VEGA database (http://vega.archive.ensembl.org/). Ensembl transcript IDs were mapped to gene symbols using the GENCODE v22 reference. DNA methylation data (Illumina HumanMethylation450 BeadChip) were also obtained from UCSC Xena. CNA segment files were obtained from FireBrowse (http://firebrowse.org/), and somatic mutation, clinicopathological, and survival data (including overall survival [OS] and progression-free survival [PFS]) were retrieved from cBioPortal (https://www.cbioportal.org/).

### 2.2 External validation cohorts

To validate the robustness of the molecular subtypes identified, we employed two independent datasets. The first was the Conway cohort (GSE120878), consisting of genome-wide methylation profiles from 89 primary invasive formalin-fixed paraffin-embedded (FFPE) melanomas. The second cohort, known as the Gide cohort, comprised transcriptomic and clinical data from 91 metastatic melanoma patients undergoing immune checkpoint inhibitor (ICI) treatment: 50 received anti-PD-1 monotherapy, while 41 were treated with a combination of anti-CTLA-4 and anti-PD-1 therapies (12).

### 2.3 Integrative clustering of multi-omics data

Five matrices (mRNA, lncRNA, DNA methylation, CNA, mutation) were constructed for the 421 TCGA samples. Expression data were log2-transformed. Using the “ChAMP” R package, methylation data were filtered to retain probes within promoter CpG islands. For multiple probes mapping to the same gene promoter, the median β value was calculated, yielding 10,270 gene-level values. Mutation status was binarized (1 = mutated nonsynonymous, 0 = wild-type). CNA features were processed according to published methods.

To ensure computational efficiency, we selected the top 500 most variable features from each data type (excluding mutations, which included 24 known melanoma driver genes) using median absolute deviation. The optimal number of clusters was identified using the clustering prediction index (CPI) and gap statistics. The “iClusterBayes” R package, which employs a fully Bayesian latent variable model, was used for integrative clustering. To address platform-specific batch effects across omics layers, ComBat normalization (sva R package) was applied to each data matrix prior to integration, following standard preprocessing procedures.

### 2.4 Tumor microenvironment inference

We utilized gene set variation analysis (GSVA) to estimate tumor microenvironment composition, employing a curated signature of 364 genes that represent 24 distinct cell types. Immune and stromal scores were calculated using the “estimate” R package. Additionally, tumor-infiltrating lymphocyte methylation (MeTIL) scores were computed for both TCGA and Conway cohorts using previously published algorithms.

### 2.5 Differential expression and pathway enrichment analysis

Differential gene expression was assessed using the “limma” R package. A gene list ranked by log2 fold change was created for gene set enrichment analysis (GSEA) using the “clusterProfiler” R package. Pathway enrichment was evaluated using Hallmark gene sets, and visualization was conducted with the “GseaVis” package (https://github.com/junjunlab/GseaVis) (13).

### 2.6 Molecular subtype characterization

Subtype-specific differences in prognosis, mutation profiles, chromosomal instability, and clinical features were characterized using the “MOVICS” R package (14). Genes with >10% mutation frequency and P < 0.05 between subtypes were considered differentially mutated. Chromosomal instability was quantified using the fraction genome altered (FGA), defined as:


R=copy number of segments/2

$$\text{FGA}={\text{B}}_{\text{r}}/\text{B}$$

Where Br is the number of bases with |log2R| > 0.2 and B is the total number of profiled bases. Focal CNAs were identified using GISTIC2.0 via GenePattern (https://www.genepattern.org/) (15), with amplification/deletion thresholds set at 0.2 and q-value < 0.05.

### 2.7 Subtype prediction in external cohorts

Nearest template prediction (NTP), a model-free classification method, was used to assign samples in external cohorts to molecular subtypes based on gene expression or methylation signatures.

### 2.8 Therapeutic response prediction

Drug sensitivity for each TCGA sample was predicted using the “pRRophetic” R package (16), based on ridge regression models trained on 727 cancer cell lines with GDSC 2016 data. Predicted IC50 values were generated for multiple chemotherapeutics. For immunotherapy, transcriptomic and response data from 47 melanoma patients treated with ICIs were analyzed (17). Subclass mapping was performed to estimate similarity between molecular subtypes and ICI responders.

### 2.9 Clinical sample collection and processing

Primary tumor and corresponding adjacent normal tissues (located more than 3 cm from the tumor margin) were obtained from five cutaneous melanoma patients undergoing surgical resection at Wenzhou Hospital of Integrated Traditional Chinese and Western Medicine from May 2022 to April 2024. Fresh specimens were snap-frozen in liquid nitrogen and stored at −80°C for future processing. The institutional ethics committee approved the study protocol, and all participants provided written informed consent.

### 2.10 RNA extraction and qRT-PCR analysis

Total RNA was extracted from tissue samples using TRIzol reagent (Invitrogen), and its purity and concentration were measured with a NanoDrop 2000 spectrophotometer (Thermo Fisher Scientific). First-strand cDNA was synthesized using the PrimeScript RT Kit (Takara) following the manufacturer’s guidelines. Quantitative real-time PCR (qRT-PCR) was performed on a QuantStudio 5 system (Applied Biosystems) using SYBR Premix Ex Taq (Takara). Relative mRNA expression levels of COL11A2 were normalized to GAPDH using the 2^−ΔΔCt method. Each reaction was conducted three times.

### 2.11 Cell culture and characterization

Human melanoma cell lines A-375 and SK-MEL-31 were sourced from authenticated providers and confirmed through short tandem repeat (STR) profiling. The cells were confirmed to be free of mycoplasma contamination. Cells were maintained in DMEM (Gibco, USA) with 10% FBS and 1% penicillin-streptomycin (Gibco) at 37°C and 5% CO_2_. For expression profiling, cells were harvested at ~80% confluence, and COL11A2 mRNA levels were quantified via qRT-PCR as described above. Each assay was conducted with three independent biological replicates.

### 2.12 siRNA transfection

Small interfering RNA (siRNA) specific to COL11A2 and a non-targeting control siRNA were synthesized and dissolved in nuclease-free water to achieve a stock concentration of 10 μM. A-375 and SK-MEL-31 cells were plated at 2 × 10^5^ cells per well in 6-well plates and incubated overnight for adherence. For transfection, 50 nM siRNA was combined with 5 μL Lipofectamine 3000 (Invitrogen, L3000015) in Opti-MEM reduced-serum medium (Gibco, 31985070) and allowed to incubate at room temperature for 15 minutes to create siRNA-lipid complexes. The complexes were gradually introduced into each well. The medium was replaced with complete growth medium after 6 hours.

Total RNA was extracted 48 hours after transfection using TRIzol reagent, then converted to cDNA and analyzed by qRT-PCR. Using the 2^−ΔΔCt method, siRNA treatment reduced COL11A2 expression by over 70% relative to the control group (p < 0.01, Student’s t-test). Each experiment was conducted using three independent biological replicates for each cell line.

### 2.13 Cell proliferation assay (CCK-8)

Post-siRNA transfection, cells were plated in 96-well plates at 3 × 10³ cells per well, in five replicates. At 24, 48, 72, and 96 hours post-transfection, 10 μL of Cell Counting Kit-8 (CCK-8) reagent (Dojindo, Japan) was added to each well and incubated at 37°C for 2 hours. Absorbance at 450 nm was recorded with a BioTek Synergy H1 microplate reader. Proliferation curves were generated based on absorbance values normalized to the 0 h time point.

### 2.14 Apoptosis assay (flow cytometry)

Apoptosis was evaluated 48 hours post-transfection. Cells were collected, rinsed twice with PBS, and stained with Annexin V-FITC and propidium iodide (PI) using the Annexin V Apoptosis Detection Kit (BD Biosciences, USA) according to the manufacturer’s guidelines. Flow cytometry analysis of stained cells was conducted immediately using a BD FACSVerse. Apoptotic populations were categorized as early apoptosis (Annexin V^+^/PI^-^) and late apoptosis (Annexin V^+^/PI^+^). Data analysis was performed using FlowJo v10.

### 2.15 Migration and invasion assays

Migration and invasion were evaluated using Transwell assays. For migration, 5 × 10^4^ cells in 200 μL serum-free DMEM were seeded into the upper chamber of Transwell inserts with 8 μm pores (Corning, USA). The lower chamber was filled with 600 μL of DMEM containing 10% FBS to serve as a chemoattractant. After incubating for 24 hours at 37°C, cells remaining on the upper surface were carefully wiped away using a cotton swab. Cells that migrated to the lower membrane surface were fixed using 4% paraformaldehyde and stained with 0.1% crystal violet. Cells were counted in five randomly selected fields under a light microscope.

For invasion assays, the Transwell inserts were pre-coated with diluted Matrigel (1:8 dilution in DMEM; BD Biosciences) and incubated for 4 hours at 37°C prior to seeding. All subsequent steps mirrored those of the migration assay.

### 2.16 Western blotting

Total protein was extracted using RIPA lysis buffer (Beyotime, China) supplemented with protease inhibitors (Roche). Protein concentrations were determined using the BCA assay (Pierce, Thermo Fisher). Equal amounts of protein (30 μg/lane) were resolved by 10% SDS-PAGE and transferred to PVDF membranes (Millipore). Membranes were blocked with 5% non-fat milk in TBST for 1 hour at room temperature, then incubated overnight at 4°C with primary antibodies: anti-cleaved Caspase-3 (1:1000, #9664), anti-E-cadherin (1:2000, #3195), anti-Bcl-2 (1:1000, #15071), anti-Vimentin (1:1000, #5741), and anti-β-actin (1:5000, #4970), all from Cell Signaling Technology. Membranes were incubated with HRP-conjugated secondary antibodies (1:5000, CST) for 1 hour at room temperature following washing. Protein bands were visualized using enhanced chemiluminescence (ECL) reagents (Millipore) and quantified using ImageJ software.

### 2.17 Statistical analyses

All statistical analyses were performed using R (v4.0.2). Two-sided tests were applied throughout. Continuous variables were analyzed using the Mann-Whitney U or Kruskal-Wallis tests, while categorical variables were assessed with Fisher’s exact test. Kaplan-Meier survival analyses used the “survminer” package with log-rank tests (18). Significance was determined by P < 0.05 or FDR < 0.05, as applicable.

## 3 Result

### 3.1 Multi-omics integrative molecular subtyping of melanoma

**Figure 1:**
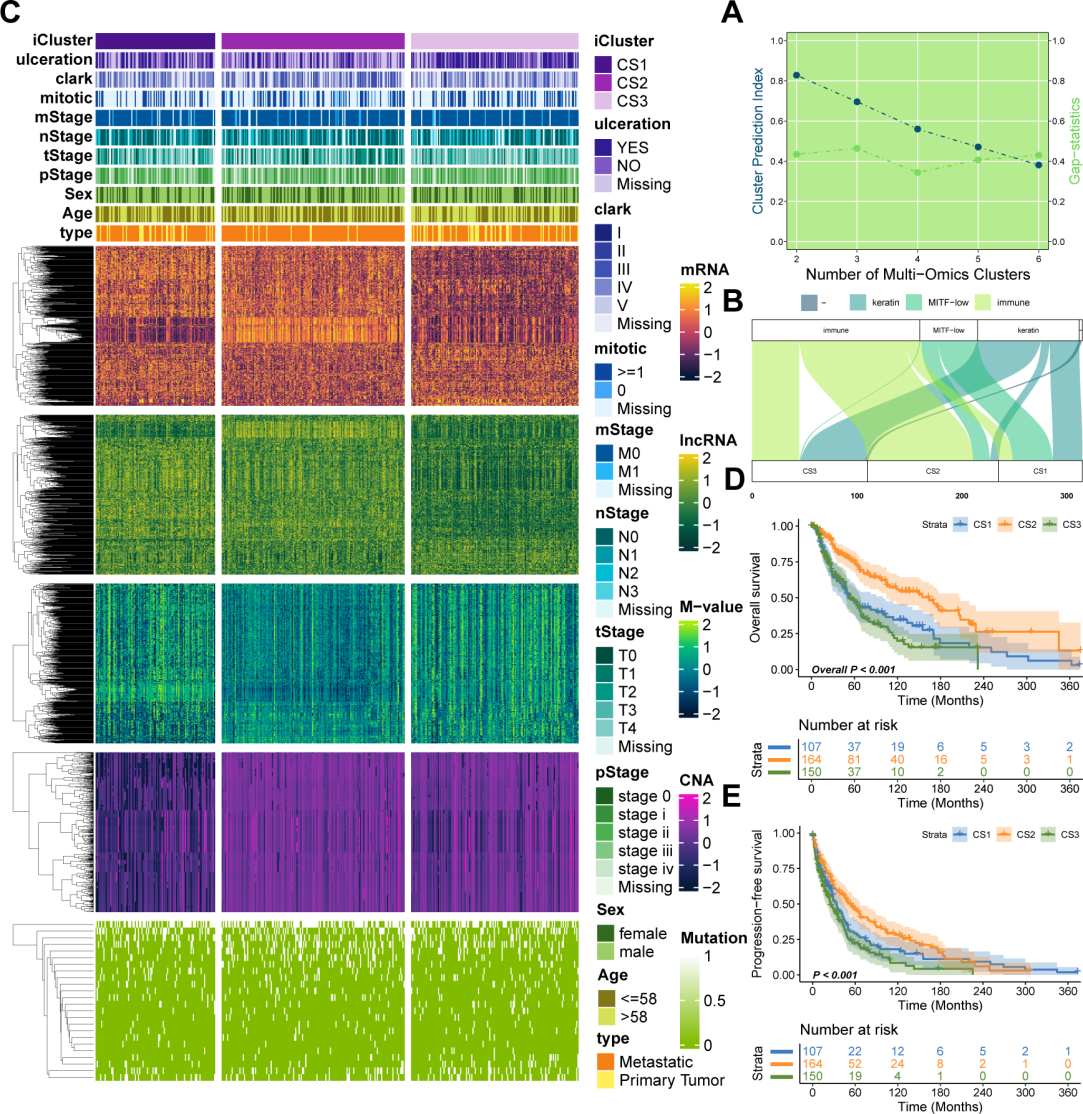
Multi-omics integrative molecular subtyping of melanoma. **(A)** Determination of the optimal clustering number based on two clustering statistics. **(B)** Integrative clustering using a fully Bayesian latent variable model identified three distinct clusters, showing overlap with previous TCGA classifications. **(C)** Distinct molecular patterns across different omics platforms: transcriptome expression, DNA methylation, CNA, and somatic mutation. **(D, E)** Kaplan–Meier survival plots showing the association of our classification with progression-free survival (PFS) and overall survival (OS).

Utilizing two clustering metrics (**Figure 1A**) and established molecular classifications for melanoma, we identified three as the optimal number of clusters. Using a fully Bayesian latent variable model to integrate five omics datasets, we discovered three distinct clusters. These clusters exhibited significant overlap with the classifications proposed by TCGA (**Figure 1B**). Importantly, our classification revealed unique molecular patterns across the transcriptome, DNA methylation, copy number alterations (CNA), and somatic mutations (**Figure 1C**). The multi-omics classification showed a strong correlation with both progression-free survival (PFS) and overall survival (OS) (P < 0.001; **Figures 1D, E**). Among the three subtypes, CS2 exhibited the most favorable prognosis. Furthermore, these subtypes were significantly associated with key clinical risk features, including T stage and pathological stage (both P < 0.05; **Supplementary Table S1**). A strong correlation was also observed between our classification and variables such as mitotic count, Clark’s level, and ulceration status (all P < 0.05; **Supplementary Table S1**).

### 3.2 Genetic dissection of integrated subtypes of melanoma

**Figure 2:**
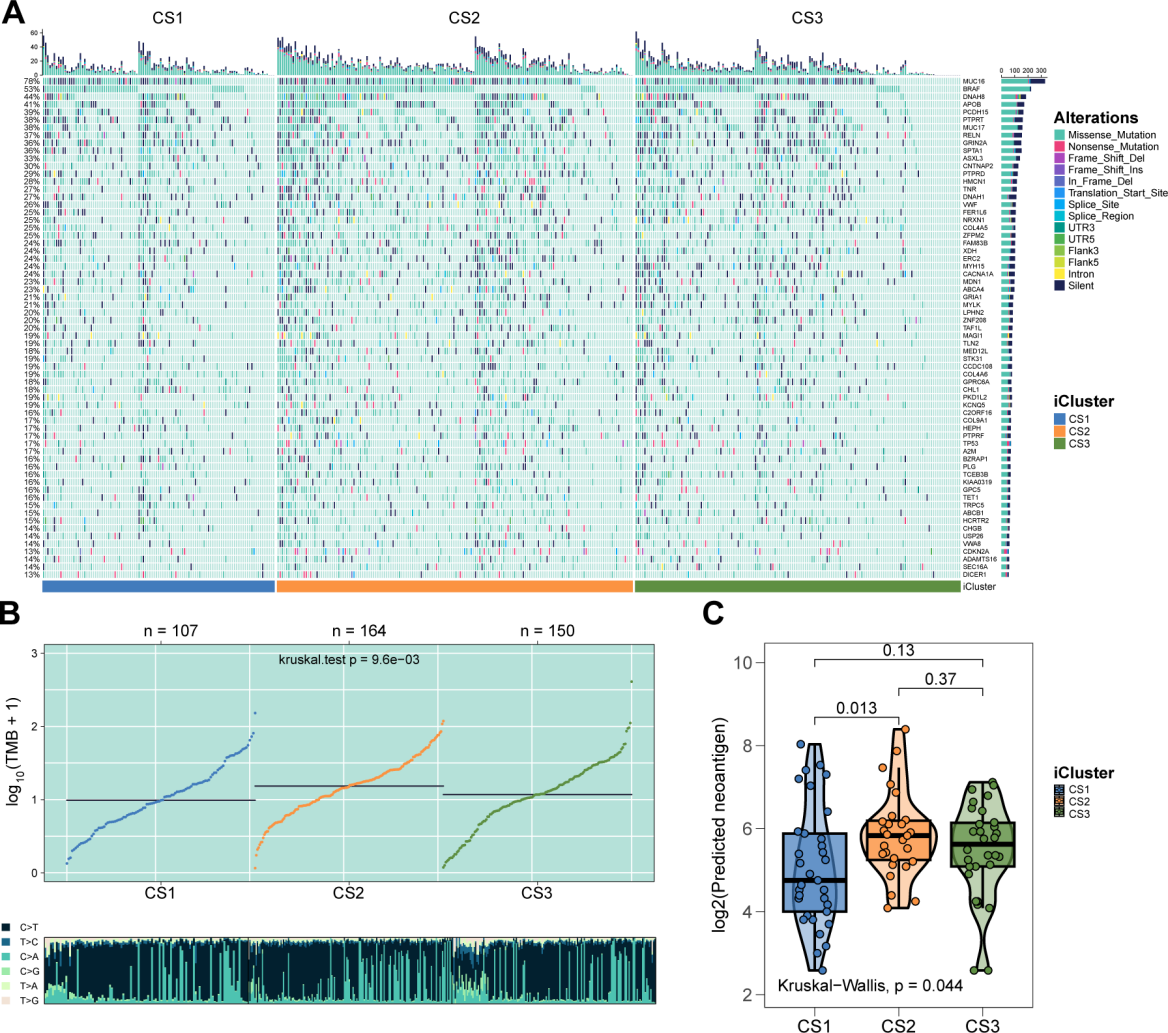
Genetic delineation of integrative subtypes. **(A)** Mutational landscape indicating 65 mutations with significantly different frequencies among the subtypes. **(B)** Box plot showing that the CS2 subtype has a significantly higher TMB. **(C)** Box plot demonstrating that CS2 has more neoantigens than CS1 and CS3.

As melanoma progresses, genetic alterations drive substantial heterogeneity. To explore the genetic distinctions among the three subtypes, we analyzed the mutational landscape of all samples, identifying 65 mutations with significantly different mutational frequencies across subtypes. These mutations were present in at least 10% of melanomas in the TCGA cohort (**Figure 2A**; P < 0.05, **Supplementary Table S2**). Among these, eight were identified as potential driver mutations for melanoma: ARID2 (17%), BRAF (52%), CDKN2A (13%), COL5A1 (23%), MECOM (22%), NF1 (17%), NRAS (28%), and TP53 (16%). Further analysis revealed that CS2 exhibited a significantly higher tumor mutational burden (TMB) compared to CS1 and CS3 (P = 0.0096; **Figure 2B**). In terms of neoantigen load, CS2 showed a significantly higher number of neoantigens than CS1 (P = 0.013; **Figure 2C**), while the difference between CS2 and CS3 was not statistically significant.

**Figure 3:**
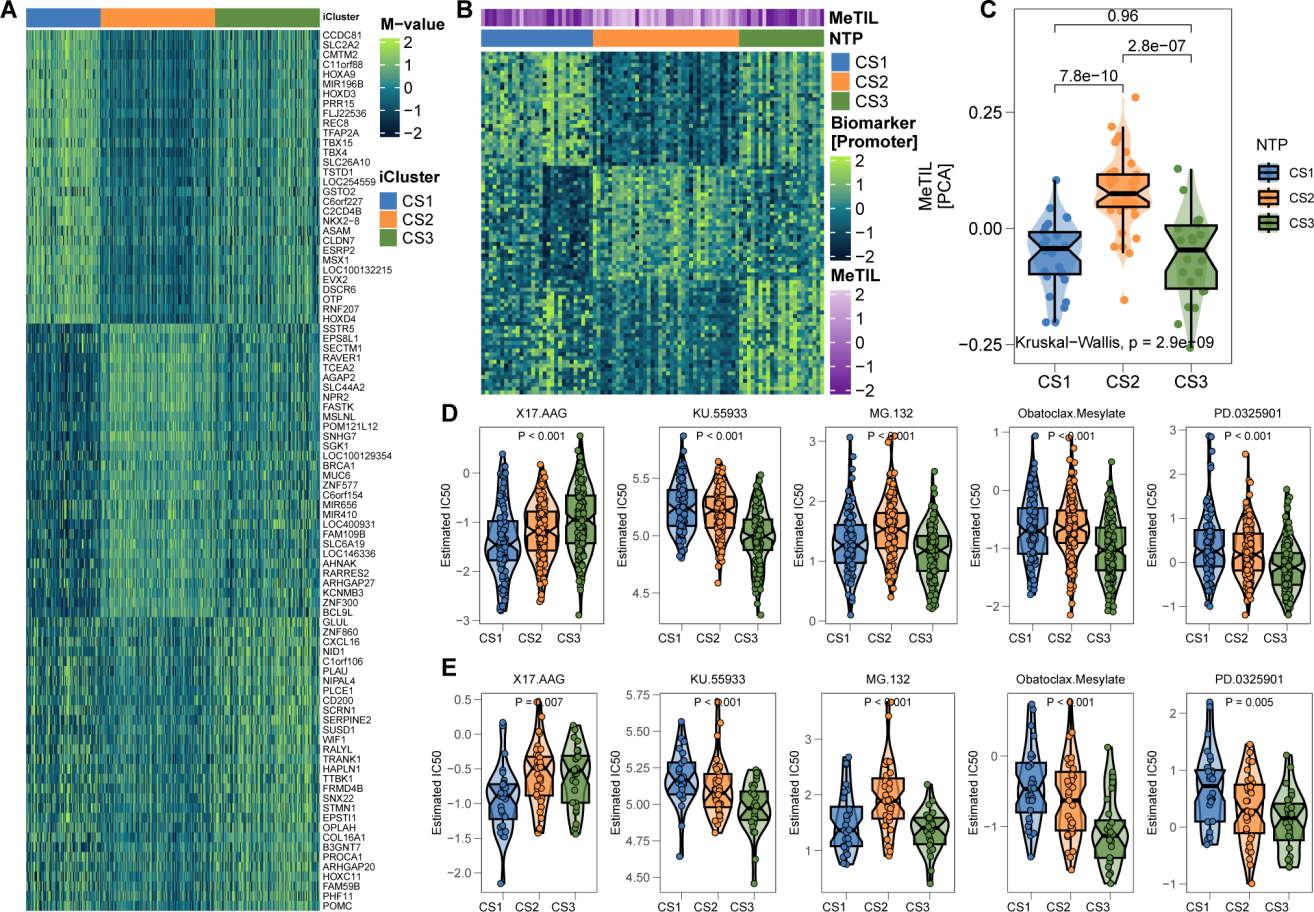
Chromosomal Instability Analysis. **(A)** Focal-level CNA profiling for each subtype. **(B)** Broad-level copy number alteration (CNA) profiling across the genome. **(C)** Quantification of chromosomal stability using FGA, FGG, and FGL metrics. **(D)** Comparative analysis showing significantly lower focal-level amplifications/deletions in CS2. **(E)** Validation of subtype-specific drug sensitivity: estimated IC_50_ distributions for five compounds across CS1–CS3 subtypes.

We next examined chromosomal instability by mapping CNAs across all human genes (**Figures 3A, B**). CS2 demonstrated superior chromosomal stability compared to other subtypes, as evidenced by lower individual fractional genome alterations (FGA) values and fewer genome gains and losses (FGG and FGL) (both P < 0.001; **Figure 3C**; **Supplementary Table S3**). Focal-level CNAs indicated that CS2 exhibited significantly fewer amplifications and deletions compared to other subtypes (P < 0.001; **Figure 3D**; **Supplementary Table S4**).

### 3.3 Differential immune profiles across melanoma subtypes

**Figure 4:**
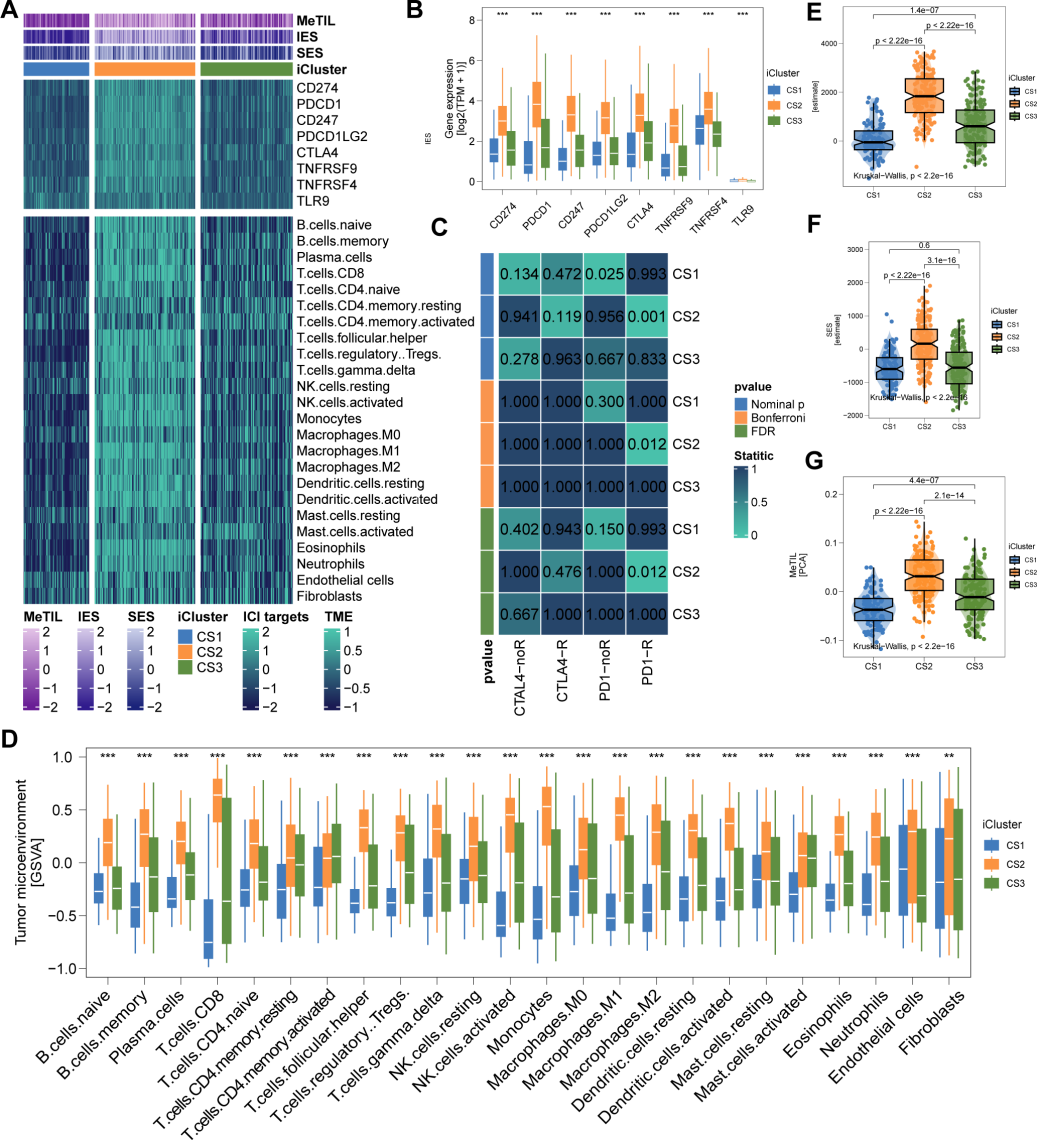
Immune Profiles across Cutaneous Melanoma Subtypes in TCGA Cohorts. **(A)** Differential immunocyte infiltration across subtypes. **(B, C)** Upregulation of immune-related genes (e.g., CD274, PDCD1, CTLA4) in CS2. **(D)** Illustration of CS2’s immune/stromal cell infiltration. **(E, F)** Bar plots showing CS2’s higher enrichment of immune and stromal cells. **(G)** Higher tumor-infiltrating lymphocyte methylation score in CS2, indicating a lower proportion of tumor-infiltrating leukocytes based on methylation. ** P<0.01, *** P < 0.001.

The genomic landscape of melanoma not only defines its molecular features but also profoundly influences its immune microenvironment. By analyzing immune cell infiltration patterns across the TCGA cohort, we observed significantly higher immune infiltration in CS2 and CS3 compared to CS1 (**Figure 4A**). Notably, CS2 exhibited elevated expression of key immune checkpoint genes, including CD274 (PD-L1), PDCD1 (PD-1), CD247 (CD3ζ), PDCD1LG2 (PD-L2), CTLA4 (CD152), TNFRSF9 (4-1BB), TNFRSF4 (OX40), and TLR9, suggesting potential susceptibility to immunotherapy (**Figures 4B, C**). Further characterization revealed extensive immune and stromal cell infiltration in CS2 (**Figures 4D-F**), which may underlie its more favorable clinical outcomes. In contrast, DNA methylation analysis indicated a lower proportion of tumor-infiltrating leukocytes in this subtype, as reflected by a significantly higher MeTIL score (P < 0.001; **Figure 4G**).

### 3.4 Validation of tumor microenvironment landscape and immunotherapy relevance

**Figure 5:**
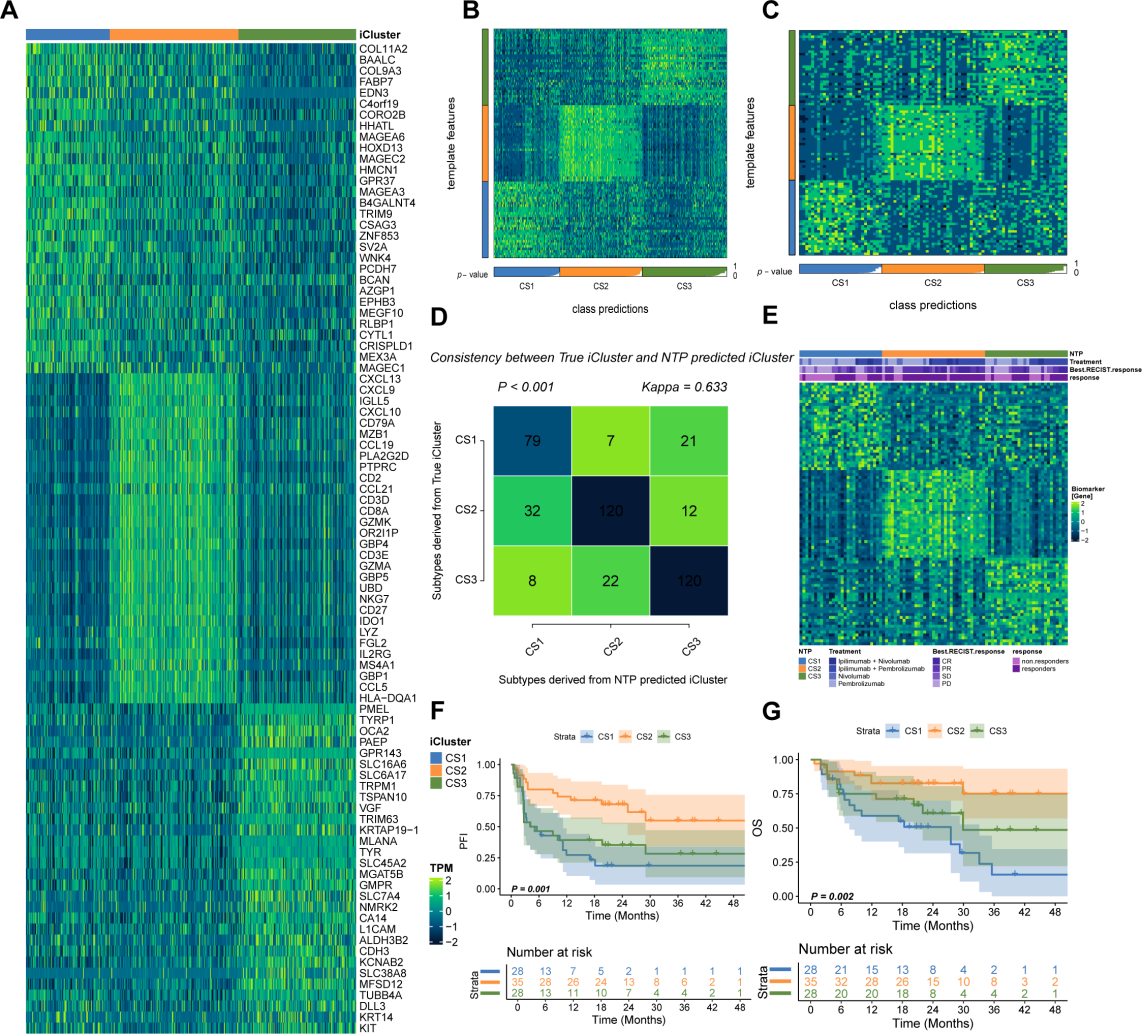
Validation in Gide’s Cohort. **(A)** Schematic of the 90-gene classifier (see **Supplementary Table S5**). **(B)** Application of the classifier in the TCGA cohort using NTP. **(C)** Classification of Gide’s cohort using the 90-gene signature. **(D)** Comparison of predicted versus actual subtype labels. **(E)** Differential immunotherapy response among predicted subtypes. **(F, G)** Kaplan–Meier plots of PFS and OS for predicted subtypes in Gide’s cohort.

**Figure 6:**
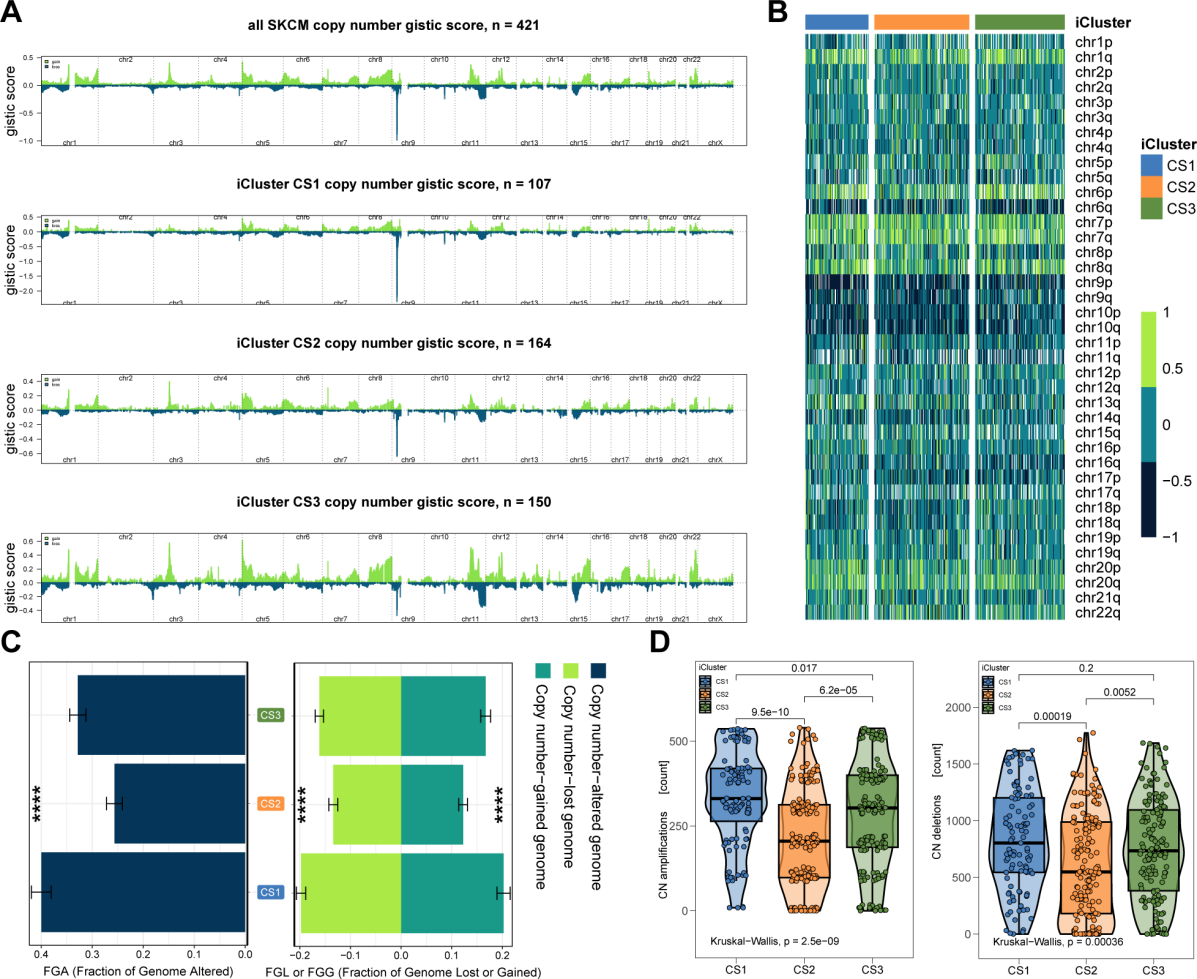
Immune Profiles across Cutaneous Melanoma Subtypes in Gide’s Cohorts. **(A)** Differential immunocyte infiltration across subtypes. **(B)** Upregulation of immune-related genes (e.g., CD274, PDCD1, CTLA4) in CS2. **(C)** Illustration of CS2’s immune/stromal cell infiltration. **(D)** Bar plots showing CS2’s higher enrichment of immune and stromal cells. **** P < 0.0001.

To test our hypothesis that CS2 may exhibit increased responsiveness to immunotherapy, we analyzed a clinical cohort of metastatic melanoma patients from Gide et al., who received either anti-PD1 monotherapy or a combination of anti-CTLA4 and anti-PD1 therapy. Using a gene-based classifier derived from the top 30 genes of each subtype, we generated a 90-gene classifier (**Figure 5A**; **Supplementary Table S5**). This classifier was applied to the TCGA cohort and demonstrated strong congruence between predicted and actual subtype labels (**Figures 5B–D**). When the classifier was applied to Gide’s cohort, we found that a higher proportion of patients predicted as CS2 responded positively to immunotherapy: 45.4% in CS1, 85.7% in CS2, and 50% in CS3 (P < 0.001; **Figure 5E**). Furthermore, patients predicted to be CS2 had improved PFS (P = 0.001; **Figure 5F**) and OS (P = 0.002; **Figure 5G**). Consistent with TCGA findings, the Gide cohort recapitulated the CS2-specific immune-enriched microenvironment, with heightened immune cell infiltration and checkpoint expression (**Figures 6A–D**).

**Figure 7:**
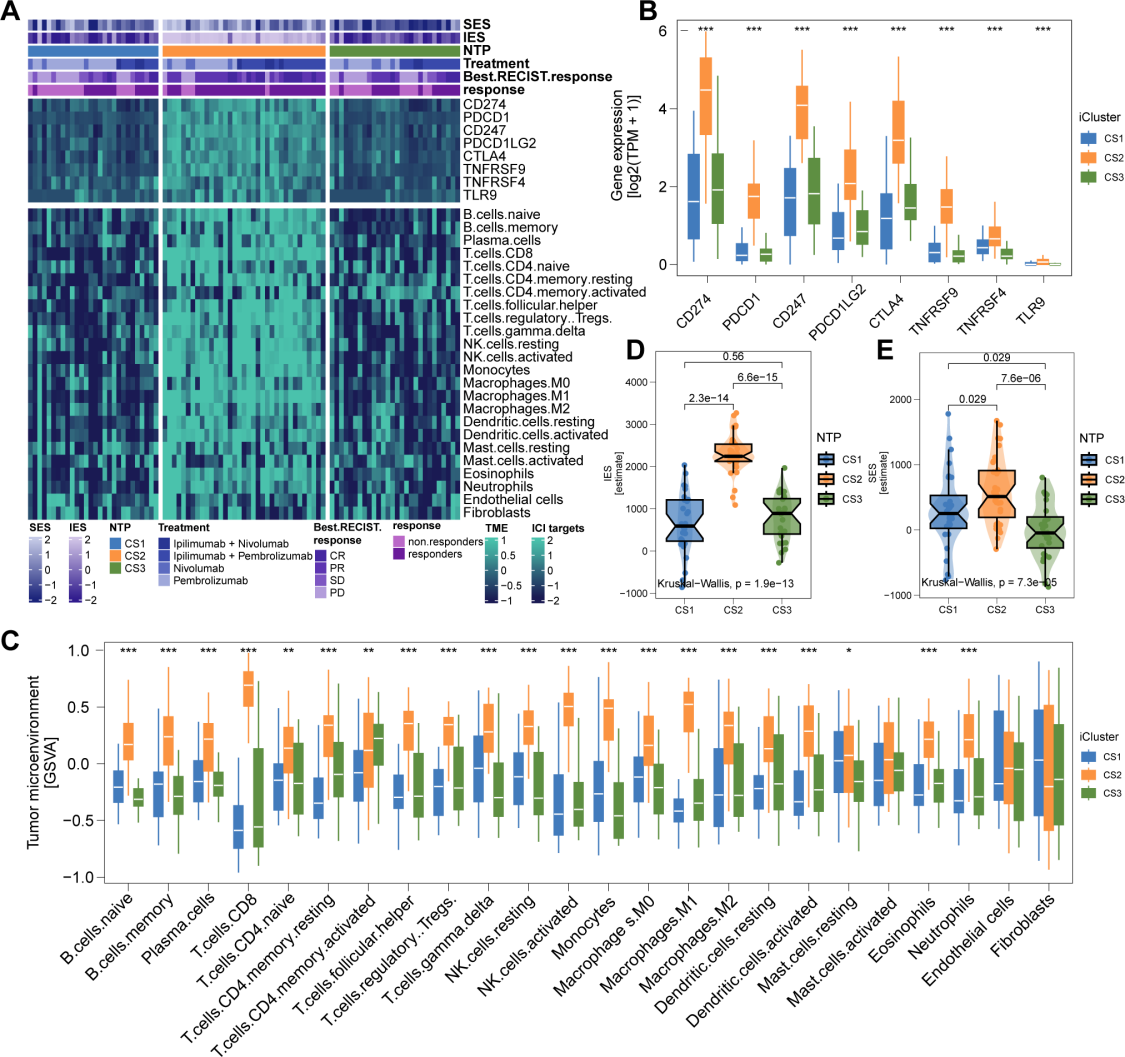
Epigenetic Validation in Conway’s Cohort and Therapeutic Drug Prediction. **(A)** 90-promoter classifier based on genes in promoter CpG islands (see **Supplementary Table S6**). **(B)** Reproduction of the three subtypes in Conway’s cohort using methylation profiles. **(C)** CS2 in Conway’s cohort shows significantly higher tumor-infiltrating lymphocyte methylation scores. **(D, E)** In silico drug sensitivity prediction using ridge regression analysis (see **Supplementary Tables S7, S8**). * P<0.05, ** P<0.01, *** P<0.001.

In further validation, we explored an epigenetic dataset of 89 primary invasive FFPE melanomas (Conway’s cohort) to assess lymphocytic infiltration via DNA methylation. A 90-gene classifier targeting promoter CpG islands was created (**Figure 7A**; **Supplementary Table S6**). Applying this classifier to Conway’s cohort successfully delineated the three subtypes (**Figure 7B**). Consistent with prior findings, CS2 exhibited significantly higher MeTIL scores (P < 0.001; **Figure 7C**), supporting the relevance of the classification system for TME analysis and immunotherapy in cutaneous melanoma.

### 3.5 Potential therapeutic strategy for melanoma subtypes

Considering the unfavorable prognosis of CS1 and CS3 melanoma patients, we aimed to identify potential therapeutic agents for these subtypes. Using an in silico drug screening approach, we built a ridge regression model to link cell line sensitivities to various compounds. This model was applied to melanoma cases from both TCGA and Gide’s cohorts with available transcriptome data. The analysis identified 17-AAG as a potential therapeutic agent for CS1, while four drugs—KU-55933, MG-132, obatoclax mesylate, and PD-0325901—were highlighted for CS3 (all FDR < 0.05; **Figures 7D, E**).

### 3.6 Functional characterization of COL11A2 reveals its oncogenic role in breast cancer cells

**Figure 8:**
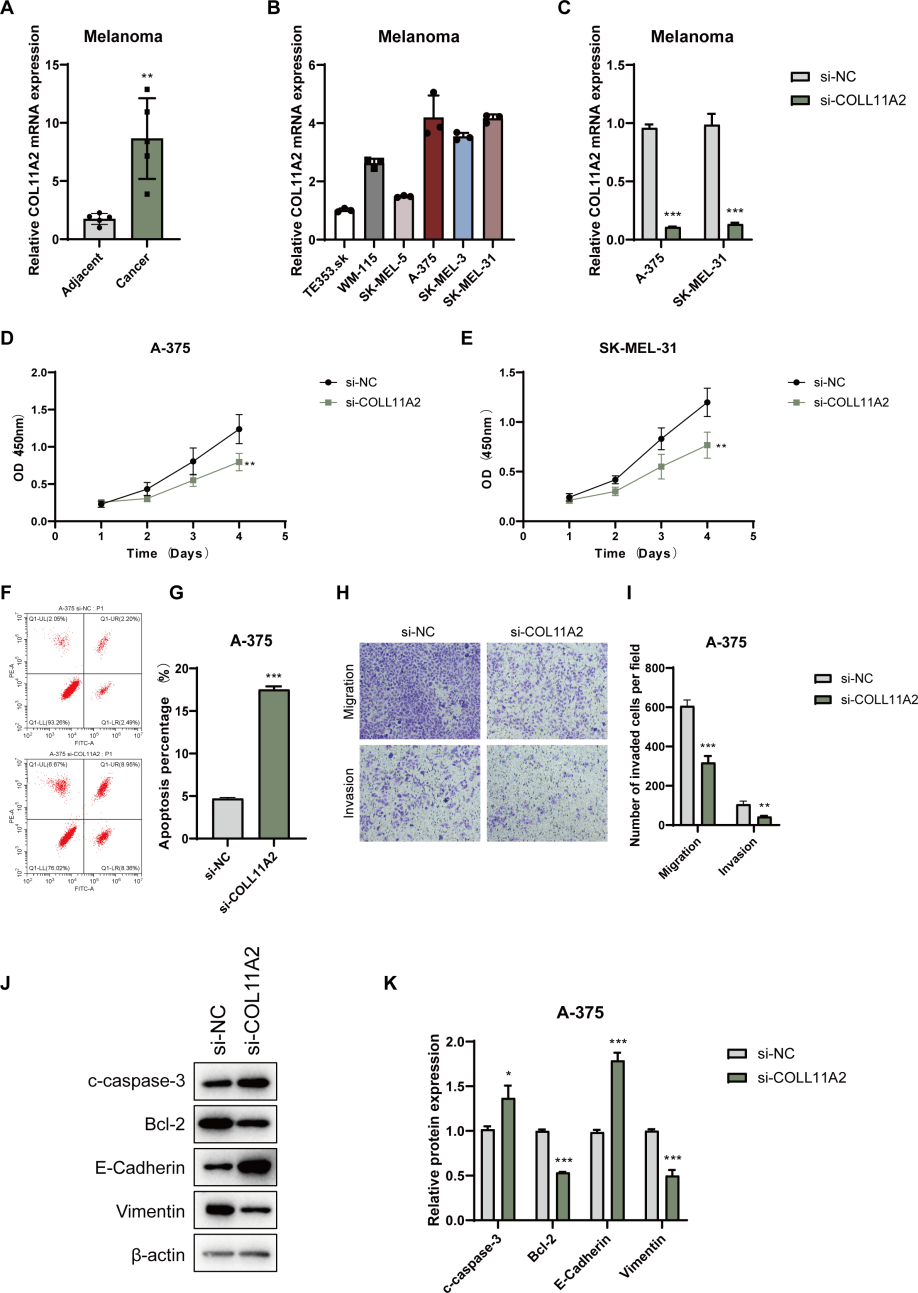
Expression and functional analysis of COL11A2 in melanoma cells. **(A)** COL11A2 expression in melanoma vs. adjacent normal tissues. **(B)** COL11A2 expression in melanoma cell lines. **(C)** qRT-PCR of COL11A2 after siRNA knockdown. **(D, E)** CCK-8 assay showing proliferation in knockdown vs. control. **(F, G)** Flow cytometry of apoptosis in A-375 cells post-knockdown. **(H, I)** Transwell assays of migration/invasion in A-375 cells. **(J)** Western blot for cleaved caspase-3, Bcl-2, E-cadherin, and Vimentin. **(K)** Relative levels of cleaved caspase-3, Bcl-2, E-cadherin, and vimentin in A-375 cells transfected with si-NC or si-COL11A2, normalized to β-actin. Data are mean ± SD (n = 3). * P<0.05, ** P<0.01, *** P<0.001.

To validate the functional significance of COL11A2, a key gene in the Prognostic Index Score (PIS) model, we investigated its expression in melanoma. Transcriptomic analysis indicated increased COL11A2 expression in melanoma tissues relative to adjacent normal tissues (**Figure 8A**), implying its role in tumorigenesis. We examined COL11A2 expression in multiple melanoma cell lines, selecting A-375 and SK-MEL-31 due to their higher expression levels (**Figures 8B, C**). Knockdown of COL11A2 via siRNA significantly reduced cell proliferation, as demonstrated by CCK-8 assays (**Figures 8D, E**). Flow cytometry revealed increased apoptosis in COL11A2-silenced A-375 cells (**Figures 8F, G**). Migration and invasion assays showed that COL11A2 knockdown impaired cell motility and invasiveness (**Figure 8H–I**). Western blot analysis demonstrated increased levels of pro-apoptotic cleaved caspase-3 (c-caspase-3) and epithelial marker E-cadherin, alongside decreased levels of anti-apoptotic Bcl-2 and mesenchymal marker Vimentin (**Figures 8J, K**), reinforcing the oncogenic function of COL11A2 in melanoma.

## 4 Discussion

In this study, we conducted an integrative multi-omics analysis of cutaneous melanoma and identified three robust molecular subtypes (CS1, CS2, and CS3), each exhibiting distinct genomic alterations, tumor microenvironmental features, and clinical outcomes. This refined molecular classification provides a comprehensive framework to decode melanoma heterogeneity and offers actionable insights into subtype-specific therapeutic vulnerabilities with potential translational relevance.

The CS2 subtype was distinguished by elevated TMB, a high neoantigen load, strong immune cell infiltration—hallmarks of an immunologically “hot” tumor microenvironment (19). Recent pan-cancer studies, including in NSCLC, have shown that copy number variation (CNV) burden—such as CNV amplitude (CNVA)—can synergize with TMB to predict immune infiltration and ICI response (20). In CS2 tumors, high CNVA correlated with increased PD-L1 expression, CD8^+^ T cell infiltration, and enhanced antigen presentation, indicating a robust, antigen-driven anti-tumor immune response. Although genomic instability is often associated with immune evasion (21), CS2 maintained high MeTIL scores, suggesting preserved immunogenicity despite elevated CNV and TMB levels. This supports the growing recognition of multidimensional biomarkers—combining TMB, CNV burden, and immune activation metrics—to more accurately predict immunotherapy responsiveness.

Notably, the coexistence of high neoantigen load and preserved immune activity in CS2 tumors raises the possibility of active immune editing as a contributing factor to their favorable prognosis. Immune editing describes a dynamic interplay in which early immune surveillance eliminates highly immunogenic clones, while selective pressure promotes the emergence of tumor cells capable of immune escape or equilibrium (22). Recent studies in melanoma have revealed that neoantigen-specific CD8^+^ and CD4^+^ T cells—including cytotoxic and regulatory subsets—can be clonally expanded in response to class I- and class II-restricted neoantigens, contributing to both immune activation and localized immunosuppression (23, 24). In CS2 tumors, sustained T cell infiltration alongside intact antigen presentation machinery may reflect an immune-edited landscape in which partial immunogenicity is retained. This evolutionary balance could account for the subtype’s robust response to ICIs, and underscores the importance of considering neoantigen quality and immune sculpting—not just quantity—in predicting therapeutic outcomes.

Furthermore, transcriptomic profiling revealed that CS2 tumors share high similarity with known ICI responders and demonstrated superior clinical outcomes in the independently validated Gide cohort. Together, these features reinforce the potential benefit of PD-1/PD-L1 blockade for this subtype and highlight the translational relevance of integrated genomic and immunologic biomarkers in stratifying melanoma patients for immunotherapy.

By contrast, the CS1 and CS3 subtypes exhibited features consistent with immune exclusion, including diminished infiltration of cytotoxic lymphocytes, high levels of chromosomal instability, and activation of oncogenic pathways associated with immune evasion. These immunologically “cold” phenotypes align with established mechanisms of immune resistance in melanoma, such as MHC class II downregulation via STAT1 silencing (25), PD-L1 induction through YAP activation in BRAFi-resistant contexts (26), and EMT-driven T cell exclusion mediated by ZEB1 (27). Additionally, tumor-derived extracellular vesicles and exosomal microRNAs have been implicated in reshaping the tumor microenvironment to suppress antitumor immunity in these settings (28). To identify potential therapeutic avenues for CS1 and CS3 tumors, we employed transcriptome-based drug response modeling, which revealed selective sensitivity to compounds including HSP90 and MEK inhibitors—agents known to counteract immune resistance by modulating tumor-intrinsic signaling and restoring immune susceptibility. Notably, HSP90 inhibitors can activate NF-κB signaling via fibroblast stimulation through extracellular vesicles (29), while MEK inhibition may reverse tumor cell dedifferentiation and synergize with BET or FAK inhibitors to overcome adaptive resistance (30). These findings underscore the potential of precision drug repositioning for targeting immunotherapy-refractory melanoma subtypes and warrant further validation in preclinical models that recapitulate both tumor cell-intrinsic and microenvironmental components.

In addition to oncogenic signaling, CS1 and CS3 tumors also exhibited distinct metabolic gene expression signatures, suggestive of subtype-specific metabolic dependencies. For example, CS1 tumors showed upregulation of FABP7, SLC2A2, and GSTO2, implicating altered lipid handling, glucose metabolism, and redox balance. CS3 tumors overexpressed GLUL, ALDH3B2, and multiple SLC family members involved in amino acid and nitrogen metabolism. Notably, recent studies have demonstrated that metabolic rewiring—including enhanced fatty acid oxidation, peroxisome function, and mitochondrial plasticity—facilitates immune evasion and MAPKi resistance in melanoma (31). These findings suggest that targeting metabolic vulnerabilities, such as glutamine metabolism or oxidative phosphorylation, may represent a complementary approach for overcoming immunotherapy resistance in CS1 and CS3 subtypes.

Functionally, we identified COL11A2 as a subtype-enriched oncogenic driver within our prognostic model. Its elevated expression was associated with unfavorable clinical outcomes and was predominantly observed in CS1 and CS3 tumors. In melanoma cells, COL11A2 silencing significantly reduced proliferation, migration, and invasion, induced apoptosis, and partially reversed EMT phenotypes. These tumor-suppressive effects align with mechanistic roles described for other fibrillar collagen family members, including COL5A1 and COL11A1, which contribute to extracellular matrix (ECM) remodeling, activation of cancer-associated fibroblasts (CAFs), and immunosuppression via stromal interactions (32–34). For instance, COL5A1 has been implicated in promoting mechanical stress and therapy resistance in lung adenocarcinoma, while COL11A1 activates CAFs through the TGF-β/NF-κB/IGFBP2 signaling axis in various solid tumors (35, 36). Given the structural and functional similarities within this collagen subfamily, COL11A2 may function analogously to promote melanoma progression by reinforcing a fibroblast-rich, immunosuppressive microenvironment. These results nominate COL11A2 as a promising therapeutic target for stromal-dominant, immune-cold melanoma subtypes and highlight the need for further validation in preclinical models that incorporate CAF dynamics and ECM remodeling.

While valuable insights were obtained, it is important to recognize certain limitations. First, the retrospective nature of multi-omics data analysis may introduce cohort-specific biases, and prospective validation in larger, independent cohorts is needed. Second, the precise molecular determinants of immune exclusion in CS1 and CS3 subtypes—particularly the contributions of epigenetic regulation, tumor cell plasticity, and stromal interactions—remain to be fully elucidated. Lastly, while our in silico drug screening provides a rational hypothesis-generating platform, experimental validation in patient-derived organoids or co-culture systems will be essential to confirm therapeutic efficacy. Additionally, tumor plasticity and potential subtype switching under therapy may affect the stability of our classification. Longitudinal studies will be needed to capture these dynamics and improve clinical utility.

## 5 Conclusion

In conclusion, we propose a novel molecular classification of cutaneous melanoma that integrates genomic, transcriptomic, and microenvironmental data to reveal biologically and clinically relevant subtypes. The CS2 subtype emerges as an immunologically favorable group likely to benefit from checkpoint blockade, whereas CS1 and CS3 subtypes may require tailored strategies targeting oncogenic pathways, stromal remodeling, and immune exclusion. Functional characterization of subtype-specific genes such as COL11A2 further supports their potential as therapeutic targets and paves the way toward personalized treatment paradigms in melanoma.

## Data Availability

The original contributions presented in the study are included in the article/**Supplementary Material**. Further inquiries can be directed to the corresponding author.

## Supplementary material

Supplementary Table 1 Associations between our classification and major clinical risk features, including T stage, pathological stage, mitotic count rate, Clark’s level, and ulceration status.

Supplementary Table 2 List of 65 mutations and their frequency among the three melanoma subtypes.

Supplementary Table 3 Broad-level CNA values and stability metrics for each subtype.

Supplementary Table 4 Focal-level CNA values and metrics for each subtype.
